# Impact of phenylalanine on cognitive, cerebral, and neurometabolic parameters in adult patients with phenylketonuria (the PICO study): a randomized, placebo-controlled, crossover, noninferiority trial

**DOI:** 10.1186/s13063-019-4022-z

**Published:** 2020-02-13

**Authors:** Roman Trepp, Raphaela Muri, Stephanie Abgottspon, Lenka Bosanska, Michel Hochuli, Johannes Slotboom, Christian Rummel, Roland Kreis, Regula Everts

**Affiliations:** 1 Department of Diabetes, Endocrinology, Nutritional Medicine and Metabolism, Inselspital, Bern University Hospital and University of Bern, Bern, Switzerland; 2 Support Center for Advanced Neuroimaging (SCAN), University Institute of Diagnostic and Interventional Neuroradiology, Inselspital, Bern University Hospital, Bern, Switzerland; 3 Magnetic Resonance Methodology Unit, Department of Biomedical Research & Institute of Interventional, Diagnostic and Pediatric Radiology, University of Bern, Bern, Switzerland; 4 Division of Neuropediatrics, Development and Rehabilitation, Children’s University Hospital, Inselspital, Bern University Hospital, Bern, Switzerland

**Keywords:** Phenylketonuria, PKU, Phenylalanine, Diet, Neuropsychology, Working memory, Neuroimaging

## Abstract

**Background:**

The population of adult patients with early-treated phenylketonuria (PKU) following newborn screening is growing substantially. The ideal target range of blood phenylalanine (Phe) levels in adults outside pregnancy is a matter of debate. Therefore, prospective intervention studies are needed to evaluate the effects of an elevated Phe concentration on cognition and structural, functional, and neurometabolic parameters of the brain.

**Methods:**

The PICO (Phenylalanine and Its Impact on Cognition) Study evaluates the effect of a 4-week Phe load on cognition and cerebral parameters in adults with early-treated PKU in a double-blind, randomized, placebo-controlled, crossover, noninferiority trial.

**Participants:**

Thirty adult patients with early-treated PKU and 30 healthy controls comparable to patients with regard to age, sex, and educational level will be recruited from the University Hospitals Bern and Zurich, Switzerland. Patients are eligible for the study if they are 18 years of age or older and had PKU diagnosed after a positive newborn screening and were treated with a Phe-restricted diet starting within the first 30 days of life.

Intervention: The cross-over intervention consists of 4-week oral Phe or placebo administration in patients with PKU. The study design mimics a Phe-restricted and a Phe-unrestricted diet using a double-blinded, placebo-controlled approach.

**Objectives:**

The primary objective of the PICO Study is to prospectively assess whether a temporarily elevated Phe level influences cognitive performance (working memory assessed with a n-back task) in adults with early-treated PKU. As a secondary objective, the PICO Study will elucidate the cerebral (fMRI, neural activation during a n-back task; rsfMRI, functional connectivity at rest; DTI, white matter integrity; and ASL, cerebral blood flow) and neurometabolic mechanisms (cerebral Phe level) that accompany changes in Phe concentration. Cognition, and structural and functional parameters of the brain of adult patients with early-treated PKU will be cross-sectionally compared to healthy controls. All assessments will take place at the University Hospital Bern, Switzerland.

**Randomization:**

Central randomization will be used to assign participants to the different treatment arms with age, sex, and center serving as the stratification factors. Randomization lists will be generated by an independent statistician.

Blinding: All trial personnel other than the statistician generating the randomization list and the personnel at the facility preparing the interventional product are blinded to the assigned treatment.

**Discussion:**

Using a combination of neuropsychological and neuroimaging data, the PICO Study will considerably contribute to improve the currently insufficient level of evidence on how adult patients with early-treated PKU should be managed.

**Trial registration:**

The study is registered at clinicaltrials.gov (NCT03788343) on the 27th of December 2018, at kofam.ch (SNCTP000003117) on the 17th of December 2018, and on the International Clinical Trials Registry Platform of the WHO.

## Background

Phenylketonuria (PKU) is a rare autosomal recessive disorder caused by a defective function of the phenylalanine hydroxylase enzyme leading to an impaired conversion of the amino acid phenylalanine (Phe) to tyrosine. Untreated PKU with increased Phe concentrations in the blood and brain during childhood leads to severe irreversible neurological impairment with, e.g., mental retardation, intellectual disability, behavioral problems, or other neurological sequelae such as epilepsy or movement disorders [1]. Newborn screening for PKU was established around 1965, enabling early treatment with a dietary restriction of Phe (low protein diet) and Phe-free protein substitutes (amino acid mixtures) soon after birth. Consequently, the population of adults with phenylketonuria with an absence of neurological disabilities has grown substantially since the early 1980s [1]. Most of these early-treated adult patients live a “normal” life as shown in an earlier study at our center, in which 15% of the adult patients with PKU went to high school or graduated from university [2].

Despite wide agreement on the treatment strategy and target Phe concentrations in childhood and for women during pregnancy, no consensus on safe Phe concentrations in adulthood has been reached so far [3]. Traditionally, the low protein diet had been enforced only during childhood and adolescence, leaving adult patients with PKU “off-diet.” Nonetheless, concerns remain that high phenylalanine levels may still have negative effects in adolescents and adults with PKU [4, 5]. Limited evidence of irreversible neurological damage exists, but over the last decade, observational and cross-sectional studies associated high Phe in early-treated adult patients with cognitive problems [6–8], psychiatric symptoms, and behavioral abnormalities [4–6, 9–13].

The executive deficit hypothesis of PKU suggests that selective impairments of executive functions may result from abnormalities of neurotransmitters, in particular dopamine, a neurochemical that is critical for prefrontal cortical functions [7]. Coming along with alterations of prefrontal cortex functions is a slight decrease in intelligence observed in patients with PKU [14]. Decreased intelligence is linked with impairments in higher-order cognitive functions, including executive functions [15] and attention [16]. In particular, various studies have reported impaired working memory performance in patients with PKU [5, 11]. Working memory is an executive function that encompasses the ability of maintaining information during a short period of time, as well as manipulating and refreshing this information to successfully complete a task [17]. Hence, working memory is crucial in everyday life; for example, it helps with focus on a demanding task or following a sequence of actions [18]. Also, other subtypes of executive functions have been reported to be affected from PKU, such as inhibition and cognitive flexibility [15]. Most of the previous studies described above, however, have focused on selected cognitive domains resulting in a lack of a comprehensive overview across cognitive domains. Such an overview is crucial to increase knowledge about the extent to which cognition is affected by PKU. More importantly, it enables a thorough evaluation of PKU treatment success.

The neurotoxicity of Phe during childhood and adolescence is additionally apparent in regard to structural and functional characteristics of the brain [19, 20]. In detail, an estimated 90% of patients with early-treated PKU display periventricular white matter lesions and reduced white matter integrity, which likely impede processing speed, often reported to be reduced in patients with PKU [21, 22]. In contrast, little is known about gray matter changes in PKU. Pérez-Dueñas and colleagues (2006) found volumetric reductions in gray matter structures including the motor cortex and thalamus [23]. However, data were acquired from a mixed sample of early- and late-treated patients, restricting the interpretation of findings. Smaller whole brain volume and smaller parietal and occipital cortex but larger volume of the putamen were described in early-treated patients with PKU [9, 19]. The latter study included patients 9–33 years old, limiting the generalizability of results due to the lack of comparability between the cognitive and neuroimaging data of children and adults. In regard to functional brain networks, atypical brain activation during a working memory task [24, 25] and decreased functional connectivity [10] have been described, likely coming along with cognitive alterations of this vulnerable patient sample. Patients with PKU additionally display neurometabolic alterations in upfield and downfield spectra, such as a decrease in choline concentration [20] and an increase in cerebral Phe level [26], respectively. Due to the limited amount of neuroimaging studies, still much uncertainty exists about the relationship between Phe concentrations and brain abnormalities in adult patients. Importantly, these studies are unable to distinguish between past effects of elevated Phe levels on the brain during childhood and adolescence and a potentially ongoing negative impact in adulthood. Previous intervention studies were mostly open label, associating the level of Phe intake in the diet with cognitive performance [27]. However, the perception of one’s own compliance with dietary recommendations may influence cognitive performance. One small intervention trial investigated the effect of the Phe concentration on cognition in a prospective randomized, placebo-controlled, double-blinded way [28]. Ten Hoedt and colleagues suggested a negative effect of high plasma Phe levels on sustained attention and on mood in nine adult patients with PKU, concluding that “a Phe restricted ‘diet for life’ might be an advisable option for many” [28].

Due to the availability of only low-grade evidence, recommendations of national and international guidelines differ substantially with regard to Phe target levels in adult patients with PKU [3, 29]. The most recent European guidelines suggest keeping Phe concentrations below 600 umol/l throughout adulthood, while stating this is a grade D recommendation. Consequently, not only are the recommendations unequally accepted by the treating metabolic specialists, but more than 50% of adults with PKU exhibit substantial difficulty in maintaining the compliance necessary to reach the recommended target Phe concentrations [30, 31]. Therefore, prospective intervention studies in adult patients with PKU are needed to evaluate the effects of dietary restrictions on cognition, cerebral and neurometabolic parameters, and quality of life.

### Objectives

The primary objective is to prospectively assess the impact of higher Phe load on cognitive performance in adults with early-treated PKU. Secondarily, the study aims to elucidate the functional and neurometabolic mechanism in the brain accompanying changes in Phe concentration.

## Methods/design

### Design

**Fig. 1:**
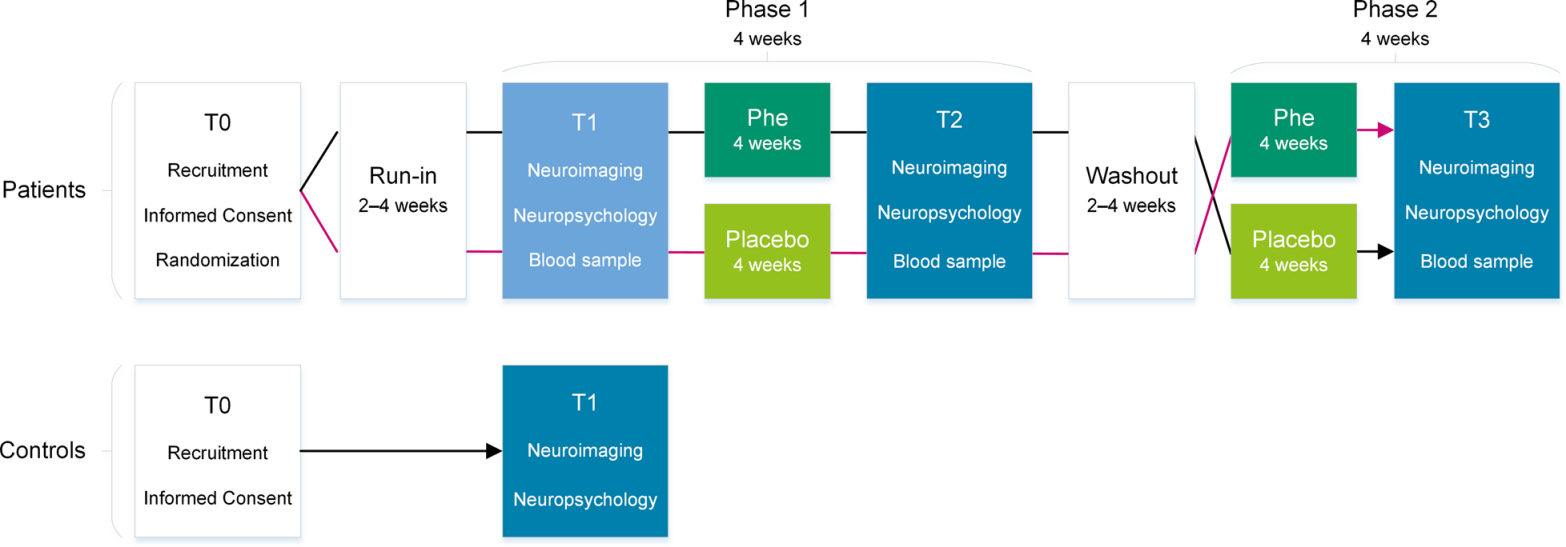
Study flow chart. Legend. T0 = screening; T1 = first assessment; T2 = second assessment; T3 = third assessment; T4 = fourth and last assessment

The PICO Study is a prospective, single-center, double-blinded, randomized, placebo-controlled, crossover, noninferiority study. In total, 30 adult patients with PKU will be included in the study. Patients with PKU will crossover, thereby serving as their own controls. To prevent the occurrence of carryover effects, a washout period of 4 weeks will be implemented in between the treatment arms (see Fig. 1). To cross-sectionally compare cognitive and neuroimaging data, 30 healthy controls will serve as a reference group.

This study protocol is written according to the SPIRIT 2013 Statement [32] providing evidence-based recommendations for the minimum content of a clinical trial protocol. SPIRIT is widely endorsed as an international standard for trial protocols. A SPIRIT Table is presented below (Table 1, Additional file 1).

**Table 1:**
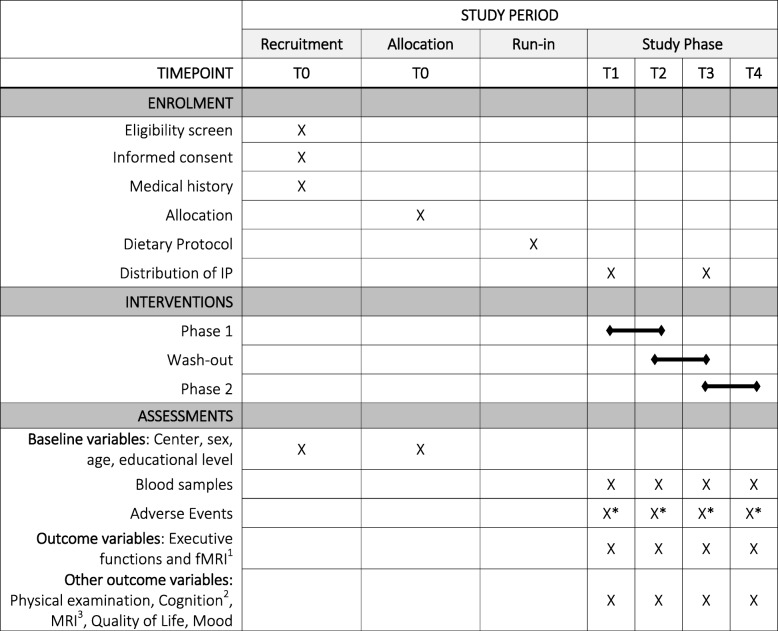
Schedule of enrolment, interventions, and assessment (according to the SPIRIT guidelines)

T0 = Recruitment, Informed Consent, Randomization; T1 = before the first treatment arm (neuropsychology, neuroimaging and blood sample assessment); T2 = after the first treatment arm (neuropsychology, neuroimaging and blood sample assessment); T3 = after the wash-out period and before the second treatment arm (neuropsychology, neuroimaging and blood sample assessment); T4 = after the second treatment arm (neuropsychology, neuroimaging and blood sample assessment); *IP* Investigational product

*additional weekly phone-calls to assess adverse events

^1^Working memory, inhibition, cognitive flexibility, neural activation during working memory fMRI

^2^IQ, memory, fine motor speed, attention

^3^Voxel-based morphometry, diffusion tensor imaging, resting-state functional imaging, arterial spin labeling, magnetic resonance spectroscopy

### Recruitment

Patients will be recruited from the treating metabolic specialist at the adult metabolic center of the University Hospital Bern and Zurich, Switzerland. Patients will be screened by the metabolic specialist either on the phone or in the course of one of their regular metabolic consultations (T0). Patients overseen at metabolic centers other than the study site will first be informed about the study by their treating metabolic specialist. If the patient is interested in the study participation, the local treating metabolic specialists informs the study physician (R.T.) who will invite the patient to the study center at the University Hospital Bern on condition that they fulfill the eligibility criteria.

Patients will be asked whether family members or friends are interested in participating in the study as control participants. Healthy controls will be additionally recruited through advertisements placed at websites, by word-of-mouth, and with flyers in the area of Bern.

Interested potential participants will be informed about the study by means of an information brochure and written informed consent will be obtained after the participants have had enough time to consider the information and to ask any questions.

### Participants

Thirty adult patients with early-treated PKU and 30 healthy controls comparable to patients with regard to age, sex, and educational level will be recruited. To achieve comparable groups, patients will be assessed first while consecutively searching for controls that are comparable in their demographics. Patients are eligible for the study if they are 18 years of age or older and have PKU diagnosed after a positive newborn screening and treated with Phe-restricted diet starting within the first 30 days of life.

Patients will be excluded if they did not follow a Phe-restricted diet within 6 months before the study, exhibited Phe concentrations above 1600 μmol/L within 6 months before the study, or have a concomitant disease status that could significantly affect primary or secondary outcomes (e.g., untreated vitamin B12 deficiency).

Female patients of childbearing potential will be excluded if they are not using or not willing to continue using at least one highly efficient method of contraception (Pearl index less than 1) for the entire study duration, are pregnant or intend to become pregnant during the course of the study, or are breast feeding.

Patients and healthy controls will be excluded if they have known or suspected noncompliance; show drug or alcohol abuse; changed medications likely to significantly interfere with cognitive functions; have known or suspected hypersensitivity or allergy to one of the ingredients of the placebo; are unable to follow the procedures of the study, e.g., due to language problems (lack of fluency in German or French); participated in another intervention study within the 30 days preceding and during the present study; or have a condition that interferes with the acquisition of MRI scans.

### Intervention

The intervention, consisting of a 4-week oral administration of Phe or placebo in patients with PKU, is initiated to simulate a controlled, temporary discontinuation of their diet. To ensure the blinded setting and avoid changing the usual low protein diet of the patients, Phe or placebo will be administered in the form of capsules. In other words, this mimics a Phe-restricted and a Phe-unrestricted diet using a double-blinded, placebo-controlled approach. Patients will be randomly assigned to one of two treatment groups starting either with Phe-containing capsules or with placebo-containing capsules. After 4 weeks, patients cross over to the alternative treatment arm. All patients and study investigators will be blinded to the treatment assignment until the completion of the study.

The investigational product (IP) consists of capsules containing 250 mg Phe and placebo capsules indistinguishable from Phe capsules and will be supplied by the Laboratorium Dr. G. Bichsel AG, 3800 Unterseen, Switzerland, according to applicable regulations. The amino acid Phe is part of the normal dietary protein and marketed as a dietary supplement, although not designated for this patient group. Using a similar approach as ten Hoedt [28], the amount of Phe (and placebo) given will be adapted according to sex and body weight with the aim to mimic the total Phe intake likely to be consumed by a healthy adult of the same sex and weight or by the patient with PKU being fully “off-diet.” Taking into account that the usual daily protein intake in adults in Switzerland is approximately 0.9–1.2 g per kilogram of body weight [33], that the average protein intake is somewhat higher in men than in women, that within a normal diet the portion of Phe of the protein content is approximately 5%, and that adult patients with mild to classical PKU mostly consume approximately 40% of the usual protein intake, the following additional doses of Phe to be administered were calculated:

- Female < 60 kg: 1500 mg per day (divided into three doses: 250 mg 2–2–2–0), ≥60 kg: 2000 mg per day (divided into three doses: 250 mg 2–2–4–0)
- Male < 60 kg: 2500 mg per day (divided into three doses: 250 mg 4–2–4–0), ≥60 kg: 3000 mg per day (divided into three doses: 250 mg 4–4–4–0)

Assignment to individual doses will be done at the first screening (T0). The assigned dose of the IP will be kept throughout the whole study, and weight fluctuations will not be considered. The capsules can be ingested before, during, or after a meal or together with the usual amino acid supplements. The last capsule of the given intervention period will be timed to be ingested with the last meal before the study visit.

To prevent the occurrence of carryover effects, a washout period of 4 weeks has been incorporated into the study design and will be implemented in between the two treatment phases (*see* Fig. 1). The bioavailability of Phe is close to 100% [34]. Phe blood concentrations are known to decrease within 1 to 2 weeks after Phe intake reduction in Phe tolerance reassessment [35]. The 4-week washout period is equivalent to 12 times the length of the estimated half-life of Phe concentration in the blood of patients, with 0% residual phenylalanine hydroxylase activity [36].

Patients will be asked to maintain their usual protein-restricted diet and amino acid supplements throughout the whole study period. In addition, patients will provide a dietary protocol during the last 3 days before the first (T1) assessment. They will be instructed to replicate the diet of the 3 days before T1 as closely as possible during the 3 days before T2, T3, and T4.

Healthy controls will not receive any intervention but will undergo the same cognitive assessment and neuroimaging measurements as patients at T1 except for blood and dry blood sampling. Healthy controls will only be assessed once (T1).

### Randomization

Computer-generated central randomization will be used to assign participants to the different treatment arms. To ensure comparability of treatment groups, the age (less than 30 years old, more than 31 years old), sex, and center (Zurich, Bern) will serve as the stratification factors. Randomization lists will be generated by an independent statistician at the Clinical Trial Unit (CTU) Bern. The randomization list will be transferred to the Laboratorium Dr. G. Bichsel AG by the independent statistician. Randomization is performed at the study site (University Hospital of Bern) by the study coordinator (S.A.), who will inform the Laboratorium about the randomization result and who will add those patient characteristics (weight, sex) that influence the IP dosage. All trial personnel other than the statistician generating the list and the personnel at the facility preparing the IP packs are blinded to the assigned treatment. In case of a severe adverse event, the treating metabolic specialist (M.H.) will first be unblinded, enabling the treatment and care of the affected patient.

### Procedure

All study visits and procedures will be performed at the study site, Inselspital, Bern University Hospital, Switzerland. In patients, cognitive performance as well as structural, functional, and neurometabolic parameters of the brain will be measured at four time points, one baseline measurement before each and one measurement after each treatment phase (see Fig. 1).

T1, T2, T3, and T4 will be carried out at the University Hospital Bern and will last approximately 5 hours (including breaks and breakfast). Each time point consists of a pregnancy test, fasting blood sample (amino acid profile), neuroimaging, cognitive assessment, physical examination, and assessment of adverse events. Cognitive assessment could be affected by drawing attention to negative influences immediately beforehand. Therefore, physical examination and assessment of symptoms and adverse effects will be placed at the end of each study visit after completion of neuroimaging and cognitive assessment.

The visits will take place in the morning. Before T2 and T4, the last IP will be taken together with the last meal, which will be placed to approach an antecedent fasting period of 10 h (range 8–12 h) before the assessment. For a given patient, T2, T3 and T4 will be scheduled for the same time of the day as the assessment at T1 to keep the length of the antecedent fasting period consistent.

#### Neuropsychology

Each of the four neuropsychological assessments (T1 to T4) will include evaluations of executive functions, namely working memory (n-back task; Test of Attentional Performance, TAP [37]; Letter-Number-Sequencing; Wechsler Adult Intelligence Scale Fourth Edition, WAIS-IV [38]), inhibition, cognitive flexibility and fluency (Color-Word Interference Test; Delis-Kaplan Executive Function System, D-KEFS [39]). Further, attention (Alertness, Sustained Attention, Divided Attention; TAP [37]), verbal and design fluency (D-KEFS [39]), and fine motor control (Purdue Pegboard [40]) will be assessed.

Psychological questionnaires will be used to assess mood (short form of the Profile of Mood States; POMS [41]), depressive symptoms (Beck’s Depression Inventory; BDI-II [42]) and health-related quality of life (PKU quality of life; PKUQOL [43]). The first neuropsychological assessment will additionally contain the assessment of general intellectual performance (short-form of WAIS-IV [38]).

#### Neuroimaging

##### Structural imaging

Anatomical MRI will be used to determine structural characteristics of the brain. All MRI images will be acquired using a 3.0 Tesla Siemens Magnetom Prisma, (Siemens Erlangen, Germany), equipped with a 64-channel head coil. Anatomical imaging will be performed using a 3-D T1 magnetization prepared rapid gradient echo (MPRAGE) sequence for acquisition of T1-weighted structural brain imaging (acquisition time TA: 4:34 min, repetition time TR = 1950 ms, echo time TE = 2.26 ms, slices per slap 176, field of view FoV 256 mm, 1 mm voxel resolution).

##### Functional imaging

Resting state fMRI (rs-fMRI) will be performed to obtain information about regional interactions (functional connectivity) between and within brain regions at rest. For the investigation of rs-fMRI, a multi-band EPI sequence from the University of Minnesota (Center for Magnetic Resonance Research), TA: 6:39 min, and flip angle 52° (avoiding rf-clipping; is in the order of the Ernst angle for TR = 1300 ms and T1 of gray matter) will be used.

Functional MRI (fMRI) of working memory will be assessed to observe the influence of Phe on the characteristics of this higher-level cognitive network. fMRI will be administered using an established paradigm assessing the visuospatial working memory network (TA = 9.52 min, TR = 1000 ms, voxel size = 2 x 2 x 2, 48 slices, slice thickness 2 mm).

Diffusion tensor imaging (DTI) will be performed to measure the integrity of white matter tracts using a Double-SE weighted q-space sequence [44] with 124 directions, slice and PE acceleration 2 and 2 resp., voxel size 2.2 mm iso, slices 56, and TA: 7:55 min.

Three-dimensional arterial spin labeling (3D-ASL) will be applied to assess cerebral blood flow. An QII FAIR 3D-ASL will be administered (TA, 4:59 min; PM, REF; Voxel size, 1.5 × 1.5 × 3.0 mm; slices per slab 40; TR = 4600 ms; TE = 16.18 ms; post-labeling (inversion time) varies depending on patient and age; bolus duration 800 ms; and inversion time 1500-2000 ms). For quantification purpose of arterial blood flow, an M0 run is added.

Magnetic resonance spectroscopy (MRS) will be utilized to capture potential alterations of brain Phe concentrations. Data will be acquired with a semi-LASER sequence [45, 46].

All MR scans will be subjected to a radiological evaluation by an experienced neuroradiologist.

To minimize head motion, a head support system consisting of two pillows positioned on each side of the head will be used. Earplugs will reduce the scanner noise.

### Primary outcome and hypothesis

The main hypothesis is that in adult patients with early-treated PKU, a 4-week period of Phe load does not decrease working memory performance measured at each of the four time points (T1 to T4) using accuracy in the visual n-back task of the TAP [37].

### Secondary outcomes

The study further aims to assess the influence of a 4-week period of Phe load on working memory performance (reaction time, visual n-back task, TAP [37]), inhibition (third condition, Color-Word Interference Test, D-KEFS [39]), cognitive flexibility (fourth condition, Color-Word Interference Test, D-KEFS [39]), intensity of neural activation during the working memory task in the MR scanner (n-back task fMRI), strength of functional connectivity between brain regions related to working memory (resting-state fMRI), and on brain Phe concentrations (MRS). All secondary outcomes will be assessed at all four time points (T1 to T4).

### Other outcomes

The PICO Study will further investigate the influence of a 4-week period of Phe load on cognitive performance, specifically on sustained and divided attention, fine motor control, verbal and design fluency, processing speed, mood (short form of the Profile of Mood States, POMS [41]; Beck’s Depression Inventory, BDI-II [42]) and health-related quality of life (PKU quality of life, PKUQOL [43]). Additionally, the influence of temporarily elevated Phe concentrations on integrity of white matter tracts (DTI) and cerebral blood flow (ASL) will be investigated.

Moreover, the study assesses differences between patients with early-treated PKU and healthy controls in regard to cognitive variables, intensity of neural activation during the working memory task, strength of connectivity between brain regions involved in working memory, and structural brain characteristics.

### Statistical analysis

The calculation of the sample size is based on the primary outcome (accuracy, visual n-back task of the TAP) for the intra-individual comparison within the different groups using a crossover design. The power analysis was done using Stata based on a paired means test. A noninferiority margin of 4% in working memory (n-back task) is regarded as clinically irrelevant. This margin is based on a study with healthy adults presenting a standard deviation of 4% in the accuracy condition of the n-back task [46]. Hence, a performance change in the n-back task of 4% or less would support our noninferiority hypothesis. According to Zimmermann and Fimm (2009), the test-retest reliability of the n-back task is 0.67 [37]. The correlation between two periods will be lower. Therefore, a correlation of 0.5 was assumed for this sample size calculation. Based on reported standard deviations in a n-back task in patients with PKU ranging from 5% to 8% (off-diet: SD = 7.6; on-diet: SD = 5.7 [27]), a standard deviation of 8% is expected (assuming that patients are off-diet during the 4 weeks Phe treatment). Based on these assumptions, the PICO Study will be able to detect noninferiority at a margin of 4%, a power of 80%, and a one-sided alpha level of 0.05, with a sample size of 26 patients. From earlier studies, including patients with PKU in Bern [47], patients with PKU are known to be highly motivated. Still, we might need to compensate for possible dropouts, which are suggested to fall between approximately 18% and 30% in other studies including patients with PKU [24, 28]; therefore, the goal has been set to 30 patients. Additionally, the effect size that would be detectable at different sample sizes and correlations for the comparison of the healthy control group and the experimental group (assuming the same number of controls and patients) was assessed. With a sample size of twice 26 participants and a power of 80%, an effect size of 0.79 could be detected at a two-sided alpha level of 0.05. To match the number of patients, the goal has been set to 30 controls.

Data will be analyzed according to the intention-to-treat (ITT) principle whereby all randomized participants will be analyzed in the randomized group regardless of any protocol violations. Moreover, data will be analyzed in the per-protocol patient set, excluding patients who did not receive the randomized treatment schedule, patients who did not comply with the intervention (i.e., unable to follow all four time points, unable to adhere to the treatment arms), or patients who violated major eligibility criteria (i.e., onset of a pregnancy, onset of a concomitant disease status that could affect primary or secondary outcome).

The primary analysis will be performed in the randomized patient group. As recommended by the CONSORT statement for noninferiority trials [48], the analysis of the primary outcome will be based on the ITT as well as per-protocol patient set. Raw data of the n-back task will be transformed into standardized values using age matched normative data from the test manual. Normality of data will be assessed via graphical representation of data. Linear mixed effects models will be used to calculate the lower one-sided 95% confidence limit of the primary outcome. If the lower limit lies above the noninferiority margin of 4% in both analysis sets, noninferiority will be claimed. The mixed effects model will contain the baseline measurements, the 4 weeks measurements, and an indicator for the treatment and period as fixed effects, and a random effect for participants. All primary and secondary continuous endpoints will be analyzed via this approach. Secondary outcomes will primarily be analyzed in the ITT patient set. Secondary outcomes will additionally be evaluated in the per-protocol patient set. Furthermore, processed neuroimaging data will be compared in a cross-sectional and longitudinal manner. Due to the expected heterogeneity of the patient sample, comparisons will be focused on intra-individual changes of cerebral metabolism and functional integrity (comparison Phe vs placebo). Differences between groups will be investigated by linear mixed models, as described above. For the comparison of baseline values between patients and controls, regular linear regression models adjusted for potential confounders will be used.

One interim analysis is planned for the reassessment of the sample size. Currently no reliable data are available on the correlation between the baseline and 4-weeks measurement and between the two different treatment periods for the n-back test. Therefore, the sample size will be re-assessed after 50% of patients to assure sufficient power. The re-assessment of the sample size will only be based on the observed standard deviations and correlations between baseline and follow-up values and between treatment periods. Observed changes within and between treatment periods will not be displayed, nor will the noninferiority margin be modified. No formal testing will take place; therefore, the alpha-level does not require adjustment.

Simple descriptive statistics will be used for evaluation of questionnaires on adverse events and mood. No formal statistical testing will be performed.

### Data management

The case report forms (CRFs) in this trial are implemented electronically using a dedicated electronic data capturing (EDC) system (REDCap™, https://www.project-redcap.org/). The EDC system is activated for the trial only after successfully passing a formal test procedure. All data entered in the CRFs are stored on a Linux server in a dedicated mySQL database. Responsibility for hosting the EDC system and the database lies with CTU Bern.

### Monitoring

On-site as well as central data monitoring will be part of the quality control activities implemented for this study. Monitoring will be performed according to a separate monitoring plan in collaboration with CTU Bern.

## Discussion

The PICO Study will help provide some much-needed answers on whether a rigorous protein-restricted diet to control Phe-levels is indispensable for adults with early-treated PKU. Using a combination of neuropsychological and neuroimaging data, the PICO Study will considerably contribute to an improvement of the currently insufficient level of evidence on how adult patients with early-treated PKU should be managed.

The double-blind, placebo-controlled design will eliminate the expectation bias of patients and physicians otherwise inherent to dietary interventions. Potentially positive effects of the recommended dietary restrictions have to be proven by means of double-blinded intervention trials and not only deduced from biased open interventions and association studies. This determination is particularly important in light of the potential risks of a lifelong low-protein diet. Similar to a strict vegan diet, a Phe-restricted diet not only lacks proteins but also many micronutrients, both of which have to be monitored and compensated with supplements [49]. In addition, the restrictive diets may be associated with a predisposition or aggravation of psychiatric problems by itself: eating disorders and obsessive compulsive disorder are four times more common in patients with PKU compared to the general population and twice as common as in patients with diabetes mellitus [50].

During the intervention phase, transient mild cognitive impairments might occur due to an increased Phe load. A similar smaller study with increased Phe load over 4 weeks suggested an impairment in sustained attention as well as lower scores in mood profile but did not report any other mild or serious adverse events [28]. Serious side effects are not to be expected. Neither is hyperphenylalaninemia in adulthood known as a cause of metabolic encephalopathy, nor is there a recognized acute decompensation of PKU [51]. The probability of irreversible adverse events during the progress of the present study is therefore extremely low. Even in adult patients with PKU not following any low protein diet for a long time, only very few cases of serious neurologic events with unlikely causal relationship (some of them probably caused by severe vitamin B12 deficiency) have been described in the literature: 11 cases of spastic paraparesis, three cases of muscle weakness/difficulty walking, and three cases of vision loss [52]. Despite not following any low protein diet for a long time, most adult patients showed improved scores for attention as well as self-reported anxiety and depression after reintroducing a low-protein diet [21, 53].

Neuroimaging has become an indispensable tool to better understand brain regions of susceptibility in metabolic disease and to assess the efficacy of dietary treatment on the brain. Clinicians can benefit from the technologic advancements in neuroimaging, allowing for improved understanding of diseases and ameliorated patient management. Only a limited number of neuroimaging studies have been conducted so far in patients with PKU. This is evident on a cross-sectional level but even more so on a longitudinal level. Hence, conclusive evidence on the influence of Phe on brain structure and function is not available with the current state of knowledge. By combining a broad range of advanced MR-techniques, the PICO Study strives to advance our understanding of the disease PKU and its impact on different properties of the brain. The additional information gained by the neuroimaging results may help to characterize subgroups of patients that benefit or do not benefit from restrictive dietary interventions.

Evaluation of patients’ perceived cognitive performance is an important part of routine patient care [29]. However, patients’ reports may be prone to a substantial expectation bias as they are usually aware of their recent compliance with dietary restrictions. This double-blinded trial offers a unique opportunity to evaluate the reliability of self-assessment of adult patients with early-treated PKU.

The PICO Study also yields an economical point of view. Phe-restricted diet, including the use of amino acid supplements and low-protein medical foods, costs approximately 20,000 CHF or USD per year, and patient. Sapropterin (Kuvan®), which can be used in a small subset of patients with BH4-responsive PKU, costs approximately 180,000 CHF or USD annually. Also pegvaliase (Palynziq®), the new enzyme substitution therapy [54–56], is estimated to cost approximately 190,000 CHF or USD per year and patient. Of note, neither sapropterin nor pegvaliase enabled clinically relevant significant improvement of cognitive outcome [22, 24, 57, 58].

Two limitations of the study inherent to the design need to be mentioned. First, being “off-diet” is mimicked, not conducted. Second, the duration of the intervention does not reflect an indefinite discontinuation of the Phe-restricted diet, and long-term effects of elevated Phe levels still remain largely unknown. The oldest early-treated patients with PKU are now only in their early 50s. This is a strong argument for keeping all patients with PKU under periodic review and making sure that even those who have stopped dietary treatment are not lost to follow-up. Carefully conducted long-term cohort studies are needed to learn more about the course of the disease as patients age. Based on the current state-of-the-art, patients and their metabolic specialists are unlikely to agree to a longer intervention. A confirmation of the hypothesized noninferiority of Phe could justify a larger long-term trial and support the willingness of patients and treating metabolic specialists to participate in such a long-term clinical trial.

In summary, this double-blinded, randomized, placebo-controlled, crossover, noninferiority trial using a combination of cognitive and neuroimaging data, will considerably contribute to improve the currently insufficient level of evidence on how adult patients with early-treated PKU should be managed.

### Trial status

The study was reviewed by the Clinical Trial Unit in Bern and was approved by the ethics committee of the canton of Bern, Switzerland, in December 2018 (2018–01609). The manuscript includes all revisions. Recruitment will start in July 2019 and will end in June 2021.

## Supplementary information

**Additional file 1.** SPIRIT 2013 Checklist: Recommended items to address in a clinical trial protocol and related documents.

## Data Availability

Not applicable.

## Abbreviations

3D-ASL: Three-dimensional arterial spin labeling
ASL: Arterial spin labeling
BDI-II: Beck’s Depression Inventory II
CRF: Case report form
D-KEFS: Delis–Kaplan Executive Function System
DTI: Diffusion tensor imaging
EDC: Electronic data capturing
EPI: Echo-planar imaging
fMRI: Functional magnetic resonance imaging
GM: Gray matter
HRQoL: Health-Related Quality of Life
IP: Investigational product
ITT: Intention to treat
MRI: Magnetic resonance imaging
MRS: Magnetic resonance spectroscopy
Phe: Phenylalanine
PKU: Phenylketonuria
PKUQOL: Phenylketonuria Quality Of Life questionnaire
POMS: Profile of Mood States
rsfMRI: Resting-state functional MRI
T: Time point
TAP: Test of Attentional Performance
WAIS-IV: Wechsler Adult Intelligence Scale Fourth Edition
