# Correction to: Impact of phenylalanine on cognitive, cerebral, and neurometabolic parameters in adult patients with phenylketonuria (the PICO study): a randomized, placebo-controlled, crossover, noninferiority trial

**DOI:** 10.1186/s13063-020-04489-7

**Published:** 2020-06-22

**Authors:** Roman Trepp, Raphaela Muri, Stephanie Abgottspon, Lenka Bosanska, Michel Hochuli, Johannes Slotboom, Christian Rummel, Roland Kreis, Regula Everts

**Affiliations:** 1 Department of Diabetes, Endocrinology, Nutritional Medicine and Metabolism, Inselspital, Bern University Hospital and University of Bern, Bern, Switzerland; 2 Support Center for Advanced Neuroimaging (SCAN), University Institute of Diagnostic and Interventional Neuroradiology, Inselspital, Bern University Hospital, Bern, Switzerland; 3 Magnetic Resonance Methodology Unit, Department of Biomedical Research & Institute of Interventional, Diagnostic and Pediatric Radiology, University of Bern, Bern, Switzerland; 4 Division of Neuropediatrics, Development and Rehabilitation, Children’s University Hospital, Inselspital, Bern University Hospital, Bern, Switzerland

**Correction to: Trials (2020) 21:178**

**https://doi.org/10.1186/s13063-019-4022-z**

Following publication of the original article [1], the authors notified us that Fig. 1 was incorrect.

The corrected Fig. 1 is presented below.

**Fig. 1:**
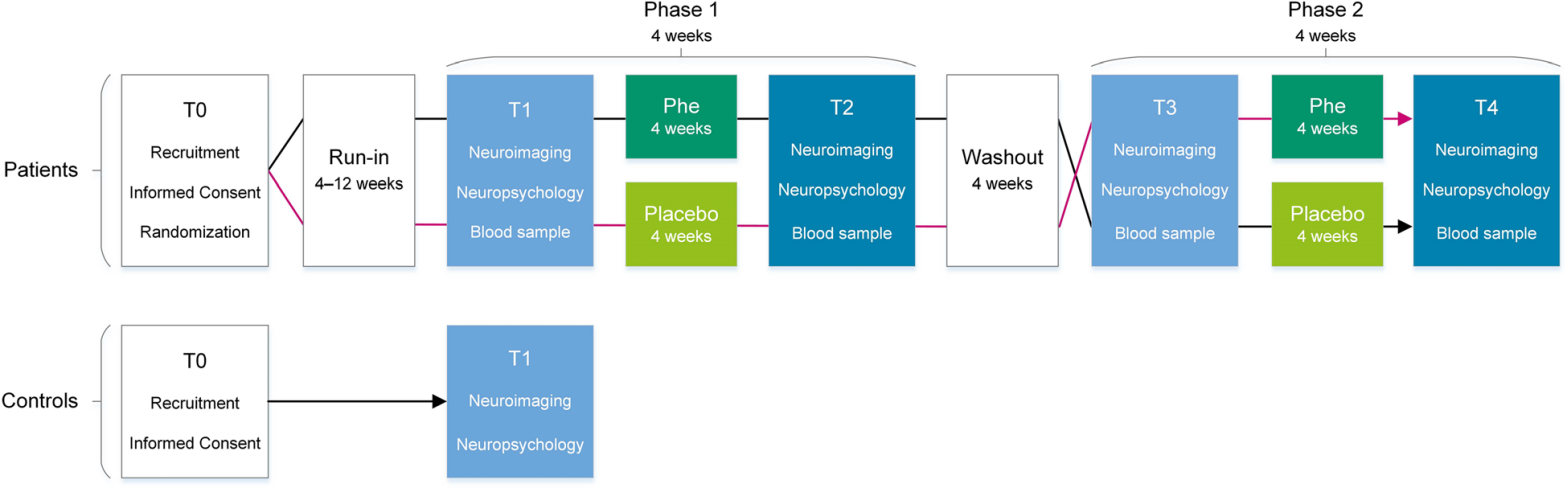
Study flow chart. Legend. T0 = screening; T1 = first assessment; T2 = second assessment; T3 = third assessment; T4 = fourth and last assessment
